# Supplementary material for: Early-life stromal niches orchestrate B lymphopoiesis at the brain’s borders
Source: bioRxiv. 2025 Nov 29:2025.11.27.690844. Preprint. [Version 1] doi: 10.1101/2025.11.27.690844 (PMC12697535; doi:10.1101/2025.11.27.690844)
Supplement: 1 [file NIHPP2025.11.27.690844V1-supplement-1.pdf]

bioRxiv preprint doi: https://doi.org/10.1101/2025.11.27.690844; this version posted November 29, 2025. The copyright holder for this preprint (which was not certified by peer review) is the author/funder, who has granted bioRxiv a license to display the preprint in perpetuity. It is made available under aCC-BY 4.0 International license.

Reagents—antibodies and RNAscope probes

Target	Fluorophore	Clone	Dilution	Manufacturer	Catalog #
αSMA	Alexa Fluor 750	1A4	100	R&D Systems	IC1420S-025
B220	Alexa Fluor 647	RA3-6B2	400	BioLegend	103229
B220	Brilliant Violet 480	RA3 6B2	100	BD Biosciences	565631
B220	Brilliant Violet 510	RA3-6B2	80	BioLegend	103248
B220	Brilliant Violet 711	RA3-6B2	160	BioLegend	103255
B220	PE/Cy5.5	RA3-6B2	320	Thermo Scientific	35-0452-80
BST-1/CD157	Brilliant Violet 421	BP-3	80	BD Biosciences	740085
BST-2	Pacific Blue	129c1	400	BioLegend	127108
CD115	Alexa Fluor 488	AFS98	400	BioLegend	135512
CD11b	Brilliant Ultraviolet 496	M1/70	100	BD Biosciences	749864
CD11b	Brilliant Ultraviolet 496	M1/70	100	Thermo Scientific	364-0112-82
CD11c	Alexa Fluor 488	N418	400	BioLegend	117311
CD11c	Brilliant Violet 510	N418	160	BioLegend	117353
CD11c	PerCP-Fire 806	N418	80	BioLegend	117376
CD127	Brilliant Violet 421	A7R34	80	BioLegend	135027
CD13	APC	QA19A79	80	BioLegend	164006
CD150	Brilliant Violet 711	TC15-12F12.2	160	BioLegend	115941
CD161/NK1.1	Brilliant Violet 650	PK136	80	BioLegend	108736
CD19	Alexa Fluor 647	6D5	400	BioLegend	115522
CD19	Brilliant Violet 510	6D5	40	BioLegend	115546
CD19	PE/Cy5.5	eBio1D3(1D3)	200	Thermo Fisher Scientific	35-0193-82
CD19	RealBlue 705	1D3 (RUO)	40	BD Biosciences	570563
CD1d	Brilliant Violet 421	1B1	160	BioLegend	123527
CD20	Brilliant Ultraviolet 737	GOT214A	40	BD Biosciences	752756
CD20	PE/Cy7	SA275A11	160	BioLegend	150420
CD200R3	Alexa Fluor 488	Ba13	400	Thermo Scientific	53-2001-82
CD200R3	RealBlue 744	Ba13	80	BD Biosciences	757549
CD23	Brilliant Ultraviolet 805	B3B4	80	Thermo Scientific	368-0232-82
CD24	Alexa Fluor 488	M1/69	1000	BioLegend	101815
CD24	Brilliant Violet 786	M1/69	80	Thermo Scientific	417-0242-82
CD3	Alexa Fluor 488	17A2	100	BioLegend	100210
CD3	Alexa Fluor 594	17A2	100	BioLegend	100240
CD3	Brilliant Violet 510	17A2	80	BioLegend	100234
CD31	Unconjugated	Polyclonal	100	R&D Systems	AF3628
CD31	Alexa Fluor 647	MEC13.3	i.v.	BioLegend	102516
CD31	Brilliant Violet 421	390	i.v.	BioLegend	102424
CD31	Brilliant Ultraviolet 805	MEC13.3	100	BD Biosciences	741939
CD34	Brilliant Violet 786	RAM34	10	BD Biosciences	742971
CD38	Brilliant Ultraviolet 395	90	200	BD Biosciences	740245
CD39	Alexa Fluor 647	Duha59	50	BioLegend	143808
CD39	PE-Cy7	Duha59	40	BioLegend	143806
CD4	Brilliant Violet 510	GK1.5	80	BioLegend	100449
CD4	Red 718	RM4-5	160	BD Biosciences	566939
CD43	APC	S7	160	BD Biosciences	560663
CD45	Alex Fluor 488	30-F11	i.v.	BioLegend	103122
CD45	Brilliant Ultraviolet 496	30-F11	200	BD Biosciences	569673
CD45	Brilliant Ultraviolet 805	30-F11	200	BD Biosciences	568336
CD45	Brilliant Violet 421	30-F11	100	BioLegend	103134
CD45	Brilliant Violet 605	30-F11	100	BioLegend	103155
CD45	Brilliant Violet 785	30-F11	100	BioLegend	103149
CD45	PE	30-F11	i.v.	BioLegend	103106
CD48	Brilliant Violet 421	HM48-1	160	BioLegend	103428
CD5	Brilliant Violet 711	53-7.3	160	BioLegend	100639
CD56	Brilliant Violet 605	809220	80	BD Biosciences	748097
CD64	Brilliant Violet 711	X54-5/7.1	100	BioLegend	139311
CD8	StarBright Blue 580	KT15	40	Bio-Rad	MCA609SBB580
CD8a	Brilliant Violet 510	53-6.7	80	BioLegend	100752
CD9	FITC	MZ3	100	BioLegend	124808
CD90.2	Alexa Fluor 488	30-H12	400	BioLegend	105316
CD90.2	Brilliant Ultraviolet 615	53-2.1	80	Thermo Scientific	366-0902-82
CD90.2	Brilliant Violet 510	30-H12	160	BioLegend	105335
CD93	PE/Cy7	AA4.1	160	BioLegend	136506
cKit	Brilliant Violet 421	2B8	100	BioLegend	105828
cKit	PE/Cy7	2B8	160	BioLegend	105814
CX3CR1	Alexa Fluor 488	SA011F11	1000	BioLegend	149022
CX3CR1	Brilliant Violet 480	Z8-50	160	BD Biosciences	567824

bioRxiv preprint doi: https://doi.org/10.1101/2025.11.27.690844; this version posted November 29, 2025. The copyright holder for this preprint (which was not certified by peer review) is the author/funder, who has granted bioRxiv a license to display the preprint in perpetuity. It is made available under aCC-BY 4.0 International license.

CX3CR1	Brilliant Violet 510	SA011F11	1000	BioLegend	149025
DLK-1	Unconjugated	Polyclonal	200	R&D Systems	AF8277
Isotype ctr. (for DLK-1)	Unconjugated	Polyclonal	200	R&D Systems	AB-108-C
ER-TR7	Alexa Fluor 405	ER-TR7	100	Novus Biologicals	NB100-64932AF405
ER-TR7	Alexa Fluor 488	ER-TR7	96	Novus Biologicals	NB100-64932AF488
EVA1	Brilliant Violet 711	G9P3-1	40	BD Biosciences	752401
F4/80	Alexa Fluor 488	BM8	100	BioLegend	123120
F4/80	Brilliant Violet 510	BM8	80	BioLegend	123135
F4/80	eFluor 570	BM8	100	Thermo Fisher Scientific	41-4801-82
FLT3/CD135	PE	A2F10	40	BioLegend	135305
GFP	Unconjugated	Polyclonal	500	Aves Labs	GFP-1020
GR-1	Alexa Fluor 488	RB6-8C5	400	BioLegend	108417
GR-1	Brilliant Ultraviolet 395	RB6-8C5	100	BD Biosciences	563849
GR-1	Brilliant Violet 510	RB6-8C5	200	BioLegend	108457
IgD	Alexa Fluor 594	11-26c.2a	100	BioLegend	405740
IgD	Alexa Fluor 647	11-26c.2a	100	BioLegend	405708
IgD	Brilliant Ultraviolet 395	11-26c.2a	100	BD Biosciences	564274
IgM	Alexa Fluor 555	Polyclonal	500	SouthernBiotech	1020-32
IgM	Brilliant Ultraviolet 395	R6-60.2	100	BD Biosciences	564025
IgM	Brilliant Violet 605	II/41	100	BD Biosciences	743325
IgM	DyLight 755	Polyclonal	500	Thermo Scientific	SA5-10155
IL-7R	Alexa Fluor 647	A7R34	100	BioLegend	135019
KI67	Alexa Fluor 488	Sol1a5	100	Thermo Fisher Scientific	53-5698-82
KI67	eFluor 570	Sol1a5	100	Thermo Fisher Scientific	41-5698-80
Ly-6C	Brilliant Violet 785	HK1.4	400	BioLegend	128041
Ly-6G	PE-CF594	1A8	80	BD Biosciences	562700
LYVE-1	Alexa Fluor 488	223322	100	R&D Systems	FAB2125G
MCAM/CD146	Brilliant Ultraviolet 395	ME-9F1	100	BD Biosciences	740330
MHC II/I-A/I-E	Spark Violet 538	M5/114.15.2	200	BioLegend	107671
MHC-II	Alexa Fluor 488	M5/114.15.2	100	BioLegend	107616
NG2	Unconjugated	EPR23976-145	500	Abcam	ab275024
NK1.1	Alexa Fluor 488	PK136	400	BioLegend	108718
NK1.1	Brilliant Violet 510	PK136	40	BioLegend	108738
P2RY12	APC	S16007D	160	BioLegend	848006
P2RY12	PE	S16007D	160	BioLegend	848004
PDGFRa	PE/Cy7	APA5	160	BioLegend	135912
PDPN	Alexa Fluor 594	8.1.1	100	BioLegend	127414
PDPN	Brilliant Violet 421	8.1.1	160	BioLegend	127423
S100A9	Alexa Fluor 647	2B10	100	BD Biosciences	565833
Sca-1	Alexa Fluor 647	D7	400	BioLegend	108118
SCA-1	Brilliant Violet 510	D7	40	BioLegend	108129
Siglec-F	Brilliant Violet 605	E50-2440	160	BD Biosciences	740388
ST2	Alexa Fluor 488	RMST2-2	400	Thermo Scientific	53-9335-82
TCR β	Brilliant Ultraviolet 563	H57-597	80	BD Biosciences	748406
TCR γδ	Alexa Fluor 488	GL3	200	BioLegend	118128
TER-119	Alexa Fluor 488	TER-119	400	BioLegend	116215
TER-119	Brilliant Violet 711	TER-119	160	BioLegend	116267
TER-119	PE	TER-119	200	BioLegend	116208
Thy1/CD90.2	Brilliant Violet 480	30-H12	160	BD Biosciences	746840
		Secondary Ab.			
Chicken IgY	Alexa Fluor 488	Polyclonal	2000	Thermo Scientific	A78948
Goat IgG	Alexa Fluor 647	Polyclonal	1000	Thermo Scientific	A32849
Rabbit IgG	Alexa Fluor 555	Polyclonal	1000	Thermo Scientific	A-31572
Rabbit IgG	Alexa Fluor 647	Polyclonal	1000	Thermo Scientific	A32795

Table S1. Antibodies used for flow cytometry and immunohistochemistry. i.v.—used for intravenous labeling. Secondary antibodies (Ab.).

bioRxiv preprint doi: https://doi.org/10.1101/2025.11.27.690844; this version posted November 29, 2025. The copyright holder for this preprint (which was not certified by peer review) is the author/funder, who has granted bioRxiv a license to display the preprint in perpetuity. It is made available under aCC-BY 4.0 International license.

Item	Vendor	Catalog #
RNAscope Multiplex Fluorescent V2 Assay	Advanced Cell Diagnostics	323100
RNAscope Probe Diluent	Advanced Cell Diagnostics	300041
ImmEdge Hydrophobic Barrier Pen	Advanced Cell Diagnostics	310018
RNAscope 4-Plex Ancillary Kit for Multiplex Fluorescent Kit v2	Advanced Cell Diagnostics	323120
HybEZ™ Hybridization System	Advanced Cell Diagnostics	321710
RNAscope 4-plex Multiplex Negative Control Probes	Advanced Cell Diagnostics	321831
Mm-Foxd1-C3	Advanced Cell Diagnostics	495501-C3
Mm-Cxcl12-C3	Advanced Cell Diagnostics	422711-C3
Mm-Cxcl12-C4	Advanced Cell Diagnostics	422711-C4
Mm-Cd19-C4	Advanced Cell Diagnostics	314711-C4
Mm-Gjb6	Advanced Cell Diagnostics	458811
Mm-Matn4-C2	Advanced Cell Diagnostics	504271-C2
Mm-Cobl-C3	Advanced Cell Diagnostics	1302841-C3
Mm-Flt1	Advanced Cell Diagnostics	415541
BaseScope Probe - BA-Mm-Cxcl12-2zz-st	Advanced Cell Diagnostics	*Custom designed*
Opal™ 520	Akoya Biosciences	FP1487001KT
Opal™ 570	Akoya Biosciences	FP1488001KT
Opal™ 620	Akoya Biosciences	FP1495001KT
Opal™ 650	Akoya Biosciences	FP1496001KT
Fisherbrand Superfrost Plus Microscope Slides	Fisher Scientific	22-037-246
Invitrogen Ambion Nuclease Free Water (not DEPC treated)	Thermo Scientific	AM9937
ProLong™ Diamond Antifade Mountant	Thermo Scientific	P36970

Table S2. Reagents for RNAscope and BaseScope assays

bioRxiv preprint doi: https://doi.org/10.1101/2025.11.27.690844; this version posted November 29, 2025. The copyright holder for this preprint (which was not certified by peer review) is the author/funder, who has granted bioRxiv a license to display the preprint in perpetuity. It is made available under aCC-BY 4.0 International license.

Supplemental Figures

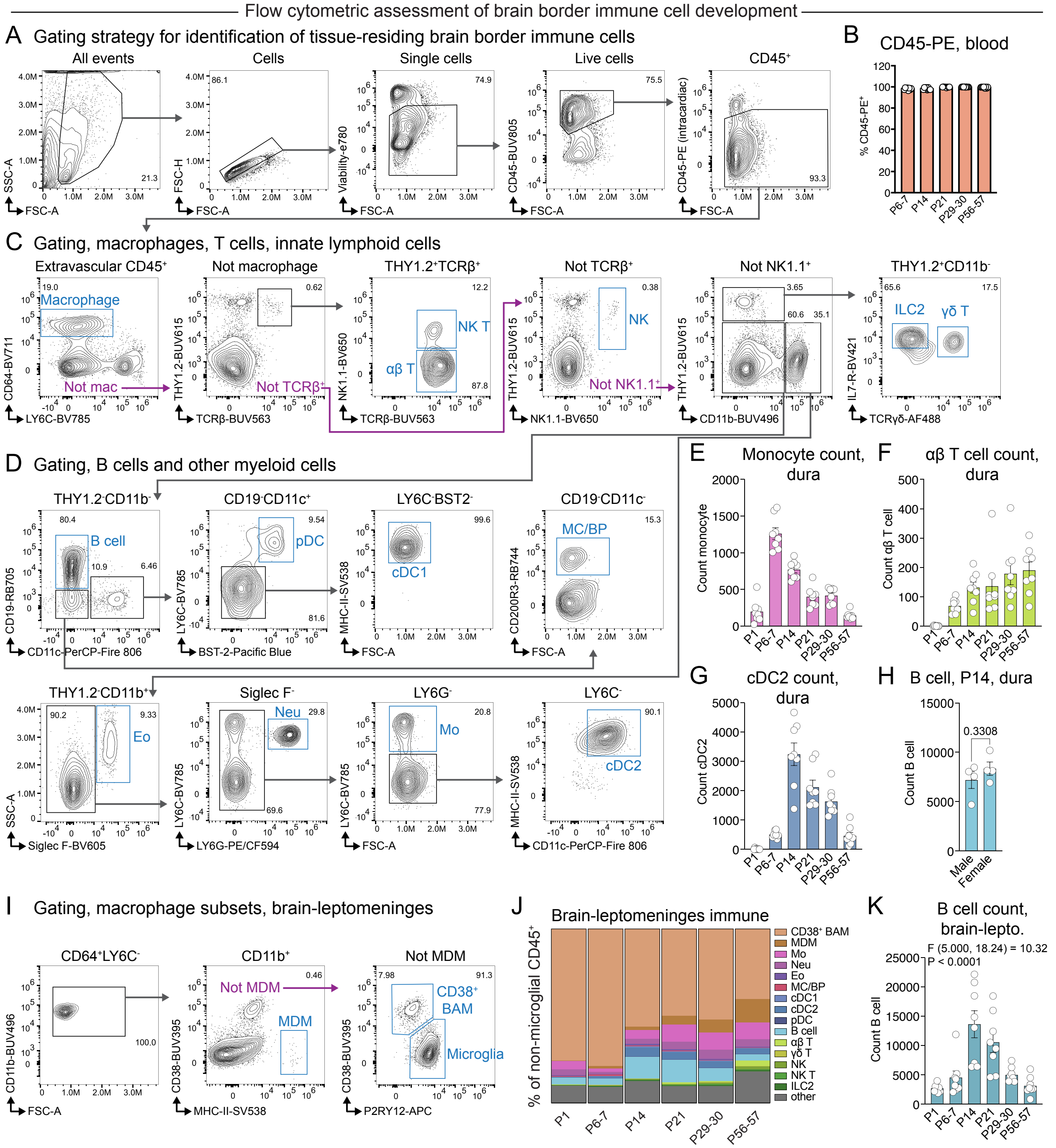

Figure S1. Temporal dynamics of brain border immune compartments in early postnatal life

(A) Flow cytometric gating strategy for assessing the composition of the dural and pooled brain and leptomeningeal immune compartments at different postnatal ages. Live, single cells were identified and then further gated for immune cells expressing CD45. Cells labeled by an intracardiac injection of CD45-PE were then excluded, identifying immune cells present in the extravascular spaces of these tissues for downstream analysis. An example from the P14 dura is shown here; other ages and brain-leptomeningeal samples were gated in a similar manner.

bioRxiv preprint doi: https://doi.org/10.1101/2025.11.27.690844; this version posted November 29, 2025. The copyright holder for this preprint (which was not certified by peer review) is the author/funder, who has granted bioRxiv a license to display the preprint in perpetuity. It is made available under aCC-BY 4.0 International license.

(B) Percentage of peripheral blood immune cells labeled by intracardiac injection of CD45-PE. n = 8 C57BL/6 mice per age pooled across two independent experiments for each timepoint. Note that P1 mice were not injected with CD45-PE due to technical limitations and are not shown here. Error bars represent SEM. Welch's ANOVA: F (4.000, 15.53) = 10.29, P = 0.0003.

(C) Gating of macrophages, T cells, and innate lymphoid cells. Extravascular $CD45^+$ immune cells were further subsetted based on combinatorial expression of surface markers. Mac—macrophage; $\alpha\beta$ T cell; NK T—natural killer T cell; NK—natural killer cell; ILC2—type 2 innate lymphoid cell; $\gamma\delta$ T cell.

(D) Gating of B cells and other myeloid cells. Cells lacking markers of macrophages, T cells, and innate lymphoid cells were further subsetted based on combinatorial expression of surface markers. $THY1.2^-CD11b^-$ cells (top row): B cells; pDC—plasmacytoid dendritic cells; cDC1—type 1 conventional dendritic cells; MC/BP—mast cells/basophils. $THY1.2^-CD11b^+$ cells (bottom row): Eo—eosinophils; Neu—neutrophils; Mo—monocytes; cDC2—type 2 conventional dendritic cells.

(E) Count of dural monocytes across postnatal ages. n = 8 C57BL/6 mice per age pooled across two independent experiments for each timepoint. Error bars represent SEM. Welch's ANOVA: F (5.000, 18.64) = 62.02, P < 0.0001.

(F) Count of dural $\alpha\beta$ T cells across postnatal ages. n = 8 C57BL/6 mice per age pooled across two independent experiments for each timepoint. Error bars represent SEM. Welch's ANOVA: F (5.000, 16.35) = 33.66, P < 0.0001.

(G) Count of dural cDC2s across postnatal ages. n = 8 C57BL/6 mice per age pooled across two independent experiments for each timepoint. Error bars represent SEM. Welch's ANOVA: F (5.000, 16.62) = 68.63, P < 0.0001.

(H) Count of dural B cells in male and female mice at P14. n = 4 C57BL/6 mice per sex pooled across 2 independent experiments for each timepoint (from the data presented in Figure 1C). Error bars represent SEM. Student's T test, unpaired, two-tailed.

(I) Subsetting of pooled brain and leptomeningeal macrophage populations. $CD64^+LY6C^-$ macrophages were subdivided into: MDM—monocyte–derived macrophages; $CD38^+$ BAMs—border-associated macrophages; and microglia.

(J) Quantification of flow cytometry data depicting immune cell proportions in the pooled brain and leptomeninges at postnatal developmental timepoints. Microglia are excluded from this analysis to allow better visualization of changes in other immune subtypes. Each value represents the mean of n = 8 C57BL/6 mice per timepoint collected across two independent experiments for each timepoint. $CD38^+$ BAM—border-associated macrophage; MDM—monocyte-derived macrophage; Mo—monocyte; Neu—neutrophil; Eo—eosinophil; MC/BP—mast cell/basophil; cDC1—type 1 conventional dendritic cell; cDC2—type 2 conventional dendritic cell; pDC—plasmacytoid dendritic cell; B cell; $\alpha\beta$ T cell; $\gamma\delta$ T cell; NK—natural killer cell; NK T—natural killer T cell; ILC2—type 2 innate lymphoid cell; other—uncategorized $CD45^+$ cells.

(K) Quantification of flow cytometry data depicting total $CD19^+$ B cell counts in pooled brain and leptomeninges (brain-lepto.) at postnatal developmental timepoints. n = 8 mice C57BL/6 mice per timepoint collected across 2 independent experiments for each timepoint. Error bars represent SEM. Welch's ANOVA.

bioRxiv preprint doi: https://doi.org/10.1101/2025.11.27.690844; this version posted November 29, 2025. The copyright holder for this preprint (which was not certified by peer review) is the author/funder, who has granted bioRxiv a license to display the preprint in perpetuity. It is made available under aCC-BY 4.0 International license.

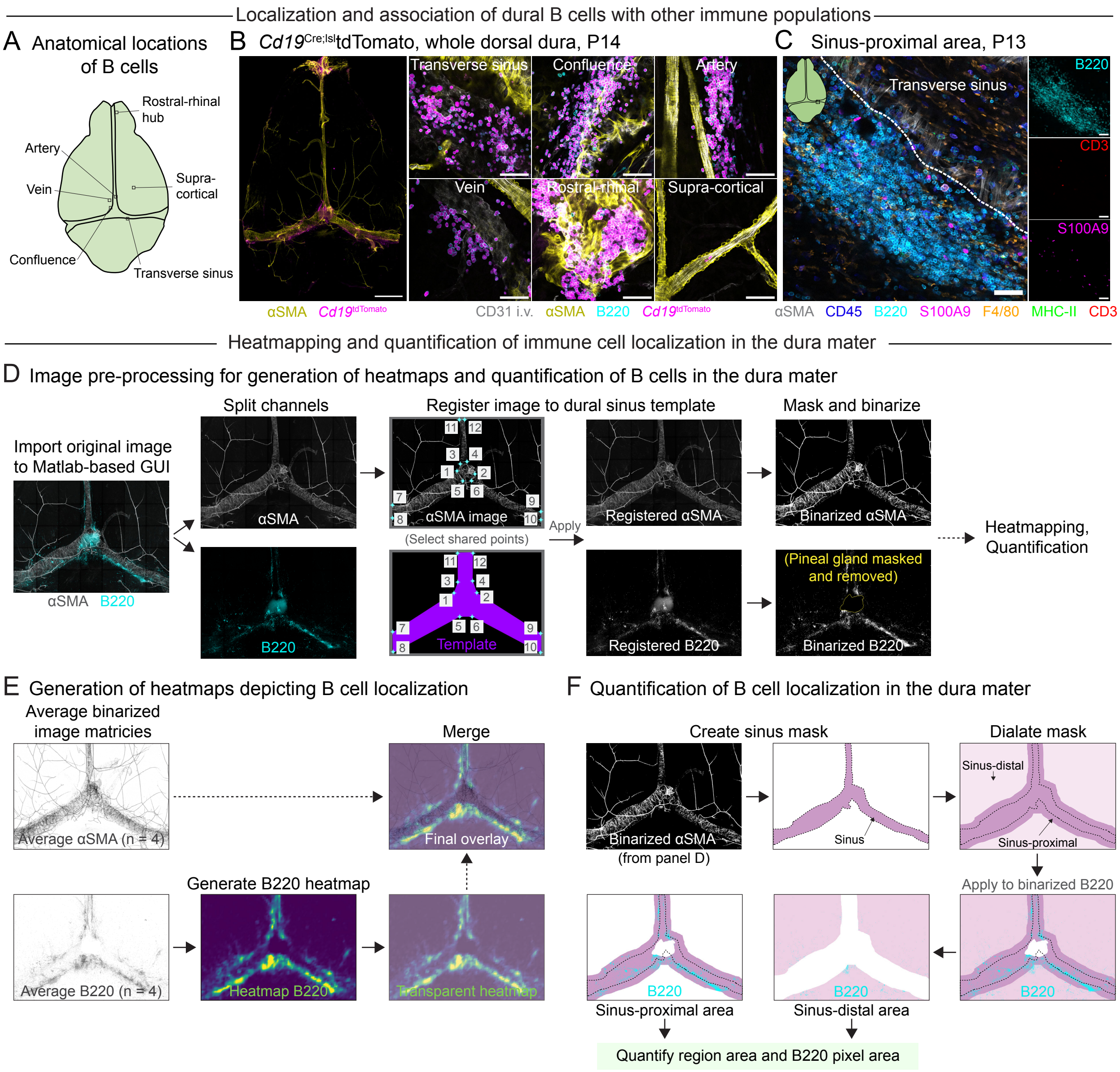

Figure S2. Spatial mapping of dural immune cells in the early-life window

(A) Diagram of a whole mount preparation of the dorsal cranial dura. Areas of interest corresponding to images in (B) are highlighted.

(B) Representative tile scan image of the dorsal dura in a P14 *Cd19*$^{Cre;lsl}$tdTomato mouse (left). High magnification images (right) depict B cell clusters in various regions of the dura. CD31 i.v.—blood vessels, white; αSMA—α-smooth muscle actin, yellow; B220—B cells, cyan; *Cd19*^{tdTomato}—B cells, magenta. Scale bar for tile scan image indicates 500 μm. Scale bars for high magnification images indicate 50 μm.

(C) Representative high magnification image of a sinus-proximal B cell cluster in the C57BL/6 mouse dura at P13. αSMA—α-smooth muscle actin, white; CD45—immune cells, blue; B220—B cells, cyan; S100A9—neutrophils, magenta; F4/80—macrophages, orange; MHC-II—antigen presenting cells, green; CD3—T cells, red. The edge of the sinus is marked with a white dotted line. Scale bars indicate 50 μm.

(D) Image pre-processing steps implemented in a custom MATLAB GUI that allows heatmapping and quantification of dural immune cells from tile scan images. Z-projected tile scan images including αSMA (structural marker of the dural sinuses) and B220 (B cell marker) were first split into individual channels. The αSMA channel was registered to a template of the dural sinuses and these registration coordinates were applied to the B220 channel, allowing

bioRxiv preprint doi: https://doi.org/10.1101/2025.11.27.690844; this version posted November 29, 2025. The copyright holder for this preprint (which was not certified by peer review) is the author/funder, who has granted bioRxiv a license to display the preprint in perpetuity. It is made available under aCC-BY 4.0 International license.

multiple images to be overlaid in the subsequent heatmapping step. Images were then binarized and autofluorescent signal from the pineal gland masked and removed before downstream processing.

(E) To generate heatmaps of B cell localization, registered, binarized images from multiple mice were merged and averaged per channel. The averaged B220 image was used to make a heatmap describing the average occurrence of B cell signal at each X-Y coordinate and overlaid with the averaged αSMA image to generate the final heatmap. The final heatmap shown here is derived from Figure 1G and is included again to allow illustration of the entire image processing pipeline.

(F) Registered and binarized images were also used to quantify the amount of B cell signal in different regions of the dura. The binarized αSMA channel (image derived from panel D) was used to generate masks of the sinus-proximal area (overlying and within 300 μm of the outer edge of the sinuses) and the sinus-distal area (> 300 μm from the edges of the sinuses). These masks were then applied to the B220 channel and used to calculate the total area of each region and the area of each region occupied by B220 signal.

bioRxiv preprint doi: https://doi.org/10.1101/2025.11.27.690844; this version posted November 29, 2025. The copyright holder for this preprint (which was not certified by peer review) is the author/funder, who has granted bioRxiv a license to display the preprint in perpetuity. It is made available under aCC-BY 4.0 International license.

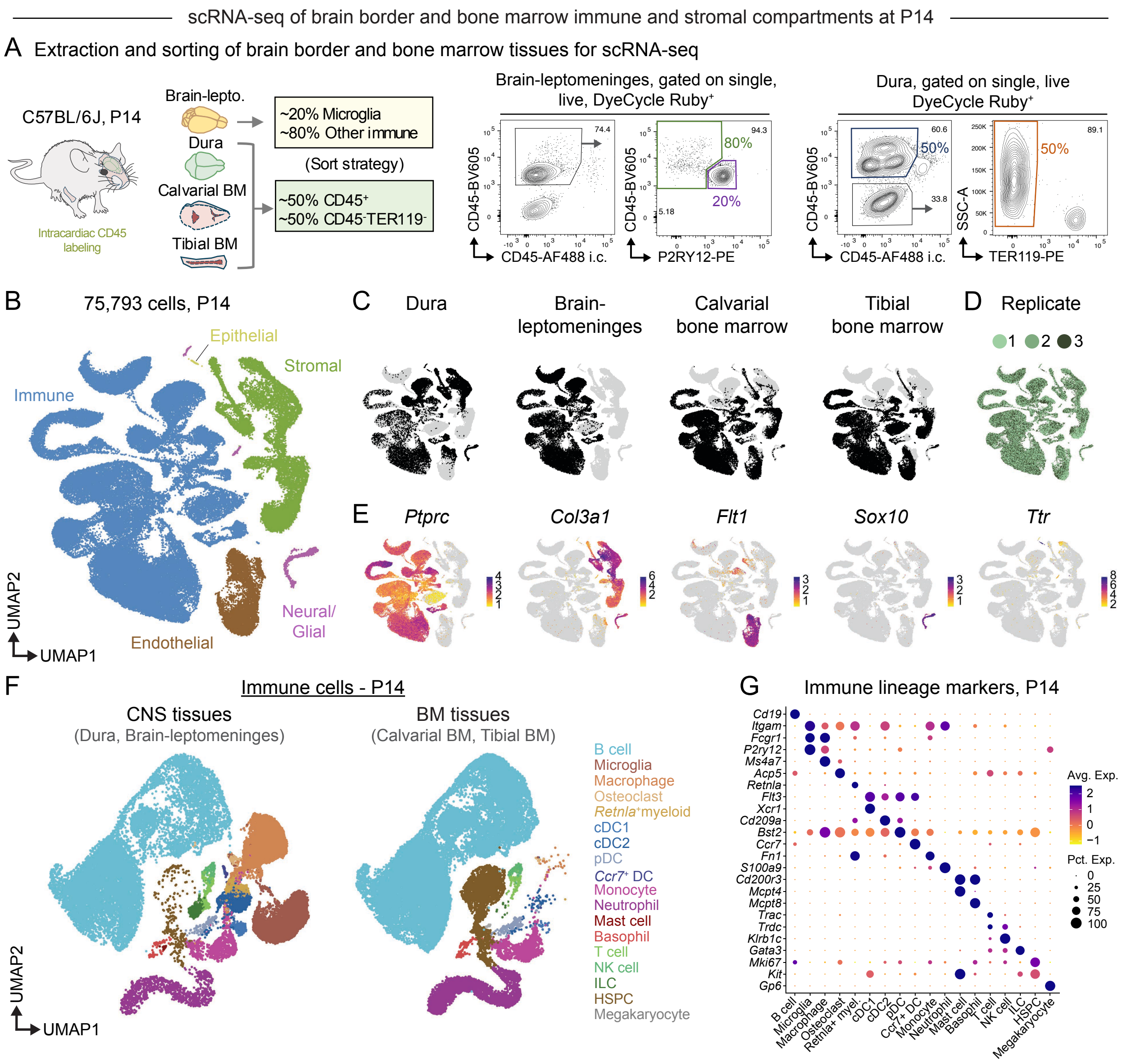

Figure S3. Molecular profiling of early-life brain border and bone marrow compartments

(A) Experimental schematic and gating strategy—fluorescence activated cell sorting of immune and non-immune cells for scRNA-seq. P14 mice were labeled intracardially (i.c) with CD45-AF488 and transcardially perfused. Dura, pooled brain and leptomeninges (Brain-lepto.), calvarial bone marrow (BM), and tibial bone marrow were extracted, digested into single cell suspensions, stained, and sorted to capture extravascular CD45$^+$ cells from all organs and CD45$^-$TER119$^-$ cells from the dura, calvarial bone marrow, and tibial bone marrow. In pooled brain and leptomeningeal samples, P2RY12$^+$CD45^{int} microglia were down sampled to allow better resolution of non-microglial cell types. Gating strategy for brain-leptomeninges: single cells, DAPI$^-$ (live), DyeCycle Ruby$^+$ (metabolically active), CD45$^+$CD45 i.c.$^-$, P2RY12$^+$CD45^{int} (microglia—sorted) and P2RY12$^-$CD45$^+$ (other immune—sorted). Gating strategy for dura, calvarial bone marrow, and tibial bone marrow: single cells, DAPI$^-$ (live), DyeCycle Ruby$^+$ (metabolically active), and then CD45$^+$CD45 i.c.$^-$ (immune—sorted) and CD45$^-$TER119$^-$ (non-immune—sorted). Cells were then captured on the Chromium 10x platform for scRNA-seq.

(B) UMAP representation of all cells across organs sampled at P14 from three male mice. Major cell classes are highlighted.

(C) Distribution of cells across organs.

(D) Distribution of cells across mouse replicates.

bioRxiv preprint doi: https://doi.org/10.1101/2025.11.27.690844; this version posted November 29, 2025. The copyright holder for this preprint (which was not certified by peer review) is the author/funder, who has granted bioRxiv a license to display the preprint in perpetuity. It is made available under aCC-BY 4.0 International license.

(E) Markers defining cell classes. *Ptprc* (CD45)—immune cells; *Col3a1*—stromal cells; *Flt1*—endothelial cells; *Sox10*—Schwann cells/myelinating cells, part of neural/glial grouping; *Ttr*—choroid plexus epithelial cells.

(F) UMAP representation of immune cells, split by CNS (dura and brain-leptomeninges) and BM (calvarial and tibial bone marrow) tissues. Cells are grouped into major immune cell types. cDC1—type 1 conventional dendritic cell; cDC2—type 2 conventional dendritic cell; pDC—plasmacytoid dendritic cell; *Ccr7*$^{+}$ DC—*Ccr7*$^{+}$ dendritic cell; NK cell—natural killer cell; ILC—innate lymphoid cell; HSPC—hematopoietic stem and progenitor cell.

(G) Marker genes for immune cell types denoted in (F).

bioRxiv preprint doi: https://doi.org/10.1101/2025.11.27.690844; this version posted November 29, 2025. The copyright holder for this preprint (which was not certified by peer review) is the author/funder, who has granted bioRxiv a license to display the preprint in perpetuity. It is made available under aCC-BY 4.0 International license.

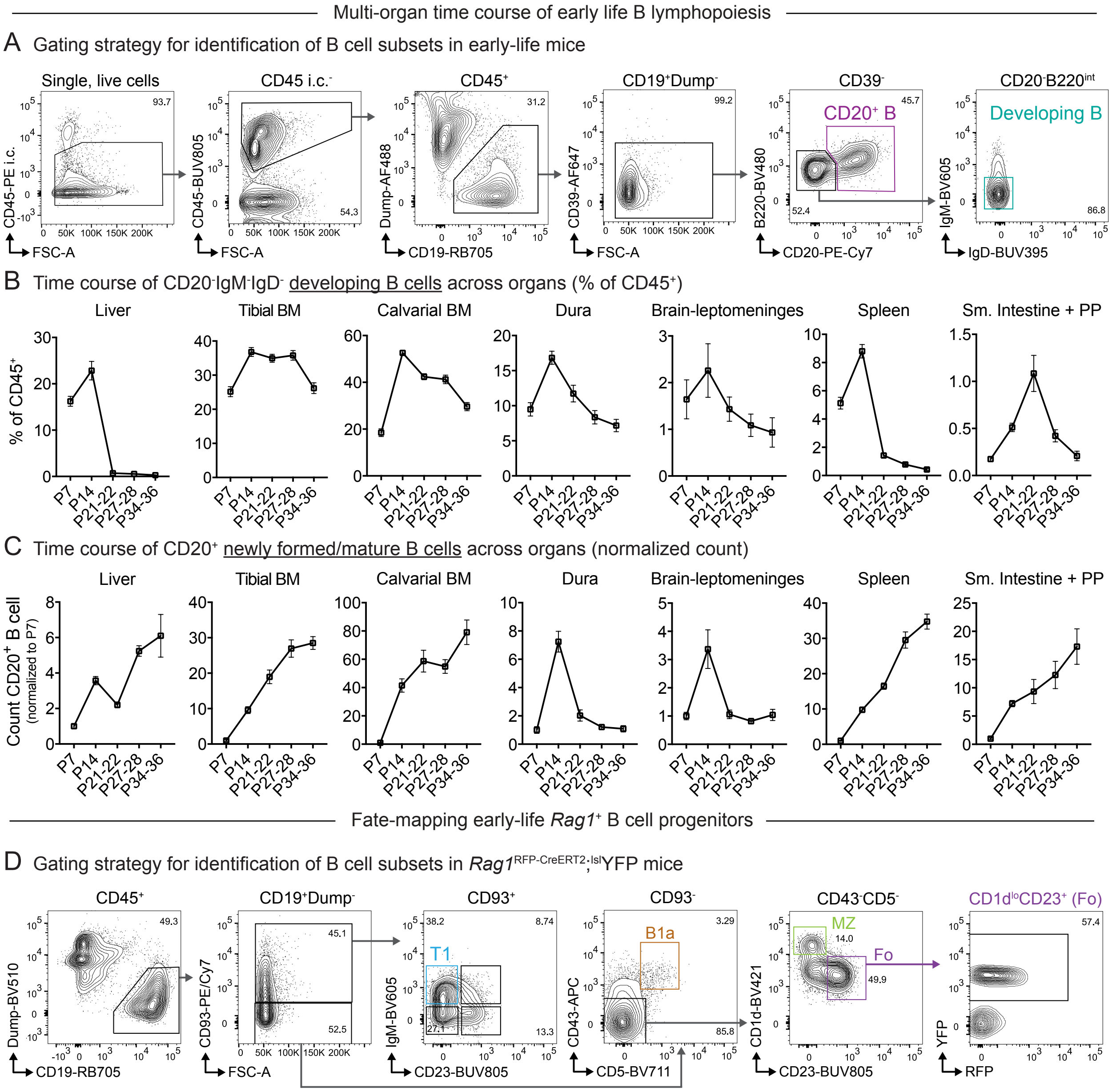

Figure S4. Mapping early life developing B cells and their contribution to the mature B cell compartment

(A) Gating strategy for identifying developing and newly formed/mature B cells over a time course of postnatal development. The P14 dura is shown and is representative of other organs and timepoints examined. B cells were gated as single, live, intracardiac (i.c.) CD45$^-$, CD45$^+$, CD19$^+$/dump channel negative (negative for GR-1, CD115, F4/80, CD11c, CD90.2, CD3, NK1.1, CD200R3, CX3CR1, TER119) cells. Plasma cells were excluded by gating for CD39^{lo} B cells, and then developing B cells identified as B220^{int}CD20$^-$, IgM$^-$IgD$^-$. Newly formed/mature B cells were identified as CD20$^+$ cells from the CD39^{lo} gate. Note that the intracardiac CD45 gate was not used for liver samples due to substantial leakage of the CD45 antibody into the tissue.

(B) Developing B cells as a percentage of CD45$^+$ cells in primary lymphoid and extramedullary organs across a time course of postnatal development, assessed by flow cytometry. n = 8 C57BL/6 mice per age at P7, P27-28, and P34-36, and 12 mice per age at P14 and P21-22, pooled from 2-3 independent experiments per age. Error bars indicate SEM. Welch's ANOVA. Liver: $F(4.000, 19.96) = 93.36$, $P < 0.0001$; Tibial bone marrow (BM): $F(4.000, 20.04) = 14.21$, $P < 0.0001$; Calvarial bone marrow: $F(4.000, 19.21) = 85.26$, $P < 0.0001$; Dura: $F(4.000, 20.67) = 15.66$, $P < 0.0001$; Brain-leptomeninges: $F(4.000, 20.35) = 1.337$, $P = 0.2902$; Spleen: $F(4.000, 20.36) = 99.28$, $P < 0.0001$; Small intestine + Peyer's patches (PP): $F(4.000, 20.14) = 15.25$, $P < 0.0001$.

bioRxiv preprint doi: https://doi.org/10.1101/2025.11.27.690844; this version posted November 29, 2025. The copyright holder for this preprint (which was not certified by peer review) is the author/funder, who has granted bioRxiv a license to display the preprint in perpetuity. It is made available under aCC-BY 4.0 International license.

(C) Normalized count of $CD20^+$ newly formed/mature B cells from primary lymphoid and extramedullary organs across a time course of postnatal development, assessed by flow cytometry. Numbers of B cells for each organ were normalized to the mean B cell count at P7. n = 8 C57BL/6 mice per age at P7, P27-28, and P34-36, and 12 mice per age at P14 and P21-22, pooled from 2-3 independent experiments per age. Welch's ANOVA. Liver: $F(4.000, 19.35) = 217.2$, $P < 0.0001$; Tibial bone marrow (BM): $F(4.000, 20.17) = 59.95$, $P < 0.0001$; Calvarial bone marrow: $F(4.000, 20.42) = 48.23$, $P < 0.0001$; Dura: $F(4.000, 20.81) = 12.81$, $P < 0.0001$; Brain-leptomeninges: $F(4.000, 20.14) = 7.723$, $P = 0.0006$; Spleen: $F(4.000, 20.12) = 138.9$, $P < 0.0001$; Small intestine + Peyer's patches (PP): $F(4.000, 19.36) = 6.115$, $P = 0.0024$.

(D) Gating strategy for identifying transitional and mature B cell subsets in *Rag1*$^{RFP\text{-}CreERT2}$;^{lsl}YFP fate mapping experiments. A representative example from the spleen is shown for the P24 harvest timepoint. B cells were identified as single, live, $CD45^+$, $CD19^+$/dump channel negative (negative for GR-1, F4/80, CX3CR1, CD11c, CD3, CD4, CD8a, NK1.1). $CD93^+$ and $CD93^-$ B cells were then separated. Transitional T1 B cells were identified from the $CD93^+$ fraction as IgM^+CD23^-. From the $CD93^-$ gate, B1a B cells were identified as $CD43^+CD5^+$ and B2 B cells were gated as $CD43^-CD5^-$. Marginal zone (MZ) B cells were identified as $CD1d^{hi}CD23^-$ and follicular B cells as $CD1d^{lo}CD23^+$. A representative example of YFP and RFP expression in splenic follicular B cells is shown. Inguinal lymph nodes were gated by the same strategy except that only follicular B cells were identified and assessed for YFP expression.

bioRxiv preprint doi: https://doi.org/10.1101/2025.11.27.690844; this version posted November 29, 2025. The copyright holder for this preprint (which was not certified by peer review) is the author/funder, who has granted bioRxiv a license to display the preprint in perpetuity. It is made available under aCC-BY 4.0 International license.

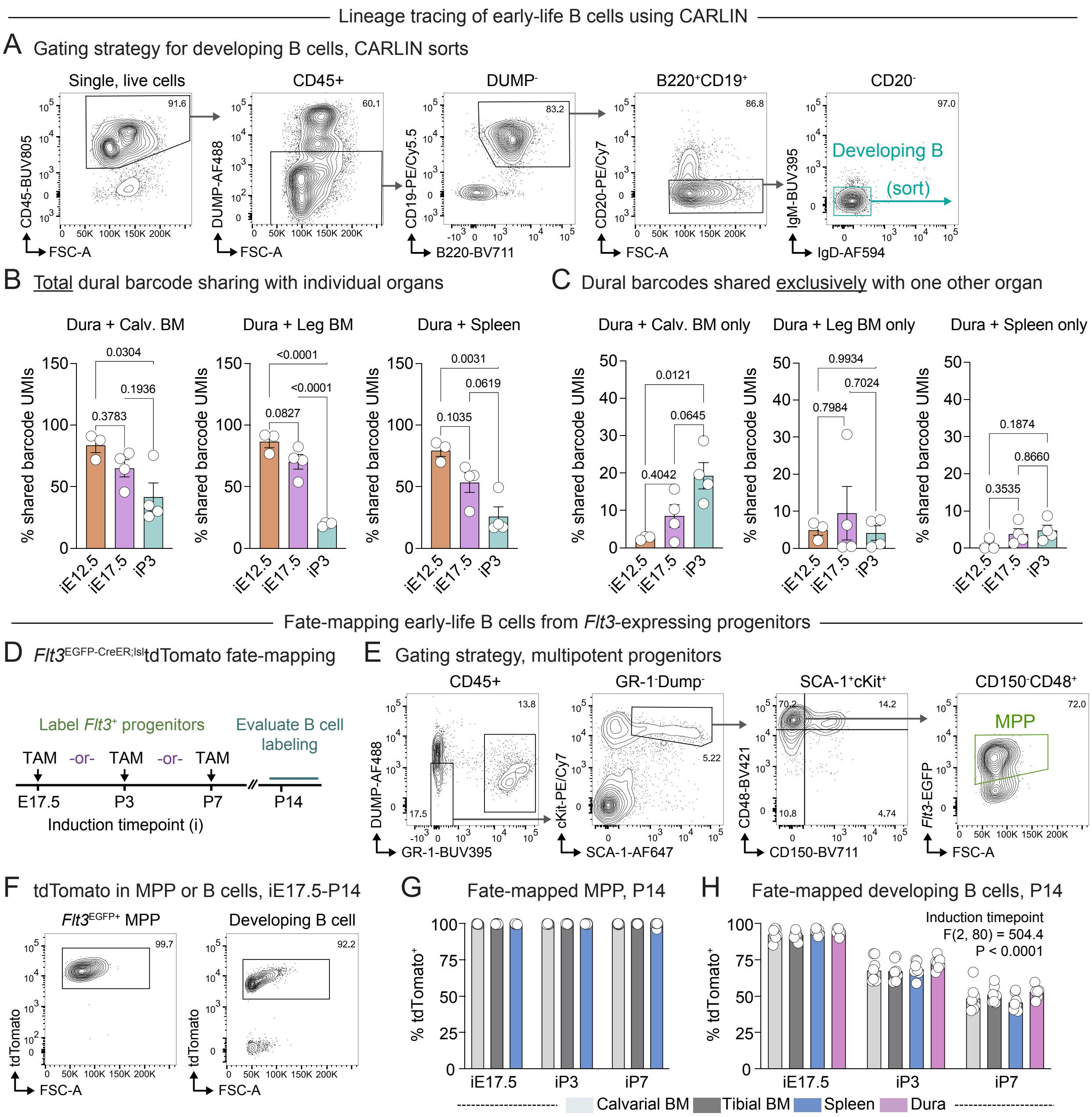

Figure S5. Lineage tracing and fate-mapping of early-life B cell progenitors

(A) Gating strategy used to sort developing B cells for CARLIN experiments. B cells were gated as live, single, CD45$^+$, Dump channel negative (negative for GR-1, F4/80, CD11c, CD3, NK1.1, CX3CR1, CD200R3, ST2, TER119), B220$^+$CD19$^+$ cells. Developing B cells (representing pooled Pro and Pre B cell stages) were further gated as CD20$^-$, IgM$^-$IgD$^-$ cells and sorted for downstream RNA sequencing. Gating strategy shown is from leg bone marrow and is representative of other organs.

(B) Total percentage of dural B cell CARLIN barcode UMIs that share an allele sequence with B cell barcodes in calvarial bone marrow (Calv. BM), leg bone marrow, or spleen. n = 3 mice at iE12.5, 4 mice at iE17.5, and 4 mice at iP3. Error bars represent SEM. One-way ANOVA with Tukey's multiple comparisons test. Dura-Calvarial BM: $F(2, 8) = 5.239$, $P = 0.0351$; Dura-Leg BM: $F(2, 8) = 61.12$, $P < 0.0001$; Dura-Spleen: $F(2, 8) = 12.01$, $P = 0.0039$.

(C) Percentage of dural B cell CARLIN barcode UMIs that share an allele sequence exclusively with B cell barcodes in one other organ. n = 3 mice at iE12.5, 4 mice at iE17.5, and 4 mice at iP3. Error bars represent SEM. One-way

bioRxiv preprint doi: https://doi.org/10.1101/2025.11.27.690844; this version posted November 29, 2025. The copyright holder for this preprint (which was not certified by peer review) is the author/funder, who has granted bioRxiv a license to display the preprint in perpetuity. It is made available under aCC-BY 4.0 International license.

ANOVA with Tukey's multiple comparisons test. Dura-Calvarial BM: $F(2, 8) = 7.913$, $P = 0.0127$; Dura-Leg BM: $F(2, 8) = 0.3816$, $P = 0.6945$; Dura-Spleen: $F(2, 8) = 1.992$, $P = 0.1985$.

(D) Experimental design for evaluating FLT3^{+} progenitor contribution to early-life B cells. *Flt3*$^{EGFP\text{-}CreER}$;^{lsl}tdTomato mice were induced with tamoxifen at iE17.5, iP3, or iP7, and then B cells evaluated at P14 for tdTomato labeling.

(E) Gating strategy for identification of multipotent progenitors (MPP) used as a reference for labeling efficiency in *Flt3*$^{EGFP\text{-}CreER}$;^{lsl}tdTomato fate mapping experiments. MPPs were identified as live, single, CD45^{+}, Dump channel negative (negative for CD19, B220, F4/80, CD11c, CD3, CD4, CD8α, NK1.1)/GR-1^{-}, SCA-1^{+}cKit^{+}, CD150^{-}CD48^{+}, *Flt3*$^{EGFP+}$. Gating shown for calvarial bone marrow and is representative of other organs.

(F) Representative flow cytometry contour plots showing tdTomato expression in multipotent progenitors (MPP) or developing B cells in calvarial bone marrow of a *Flt3*$^{EGFP\text{-}CreER}$;^{lsl}tdTomato mouse induced at iE17.5 and evaluated at P14. MPPs were gated as shown in (E). Developing B cells were gated as CD45^{+}, CD19^{+}Dump channel-negative (negative for GR-1, CD11c, CD3, CD4, CD8a, NK1.1), CD20^{-}, IgM^{-}IgD^{-}, representing Pro and Pre B cell populations.

(G) Percentage of tdTomato^{+} MPPs from calvarial bone marrow, tibial bone marrow, or spleen in *Flt3*$^{EGFP\text{-}CreER}$;^{lsl}tdTomato mice induced at embryonic or postnatal timepoints and evaluated at P14. n = 7 mice from iE17.5, 8 mice from iP3, and 8 mice from iP7 pooled from two independent experiments for each induction timepoint. Error bars represent SEM. Two-way ANOVA. Induction timepoint: $F(2, 60) = 0.6248$, $P = 0.5388$; Tissue: $F(2, 60) = 1.723$, $P = 0.1873$; Interaction: $F(4, 60) = 1.135$, $P = 0.3488$.

(H) Percentage of tdTomato^{+} developing B cells from calvarial bone marrow, tibial bone marrow, spleen, or dura in *Flt3*$^{EGFP\text{-}CreER}$;^{LSL}tdTomato mice induced at embryonic or postnatal timepoints and evaluated at P14. n = 7 mice from iE17.5, 8 mice from iP3, and 8 mice from iP7 pooled from two independent experiments for each induction timepoint. Error bars represent SEM. Two-way ANOVA. Induction timepoint: $F(2, 80) = 504.4$, $P < 0.0001$; Tissue: $F(3, 80) = 2.642$, $P = 0.0549$; Interaction: $F(6, 80) = 1.072$, $P = 0.3861$.

bioRxiv preprint doi: https://doi.org/10.1101/2025.11.27.690844; this version posted November 29, 2025. The copyright holder for this preprint (which was not certified by peer review) is the author/funder, who has granted bioRxiv a license to display the preprint in perpetuity. It is made available under aCC-BY 4.0 International license.

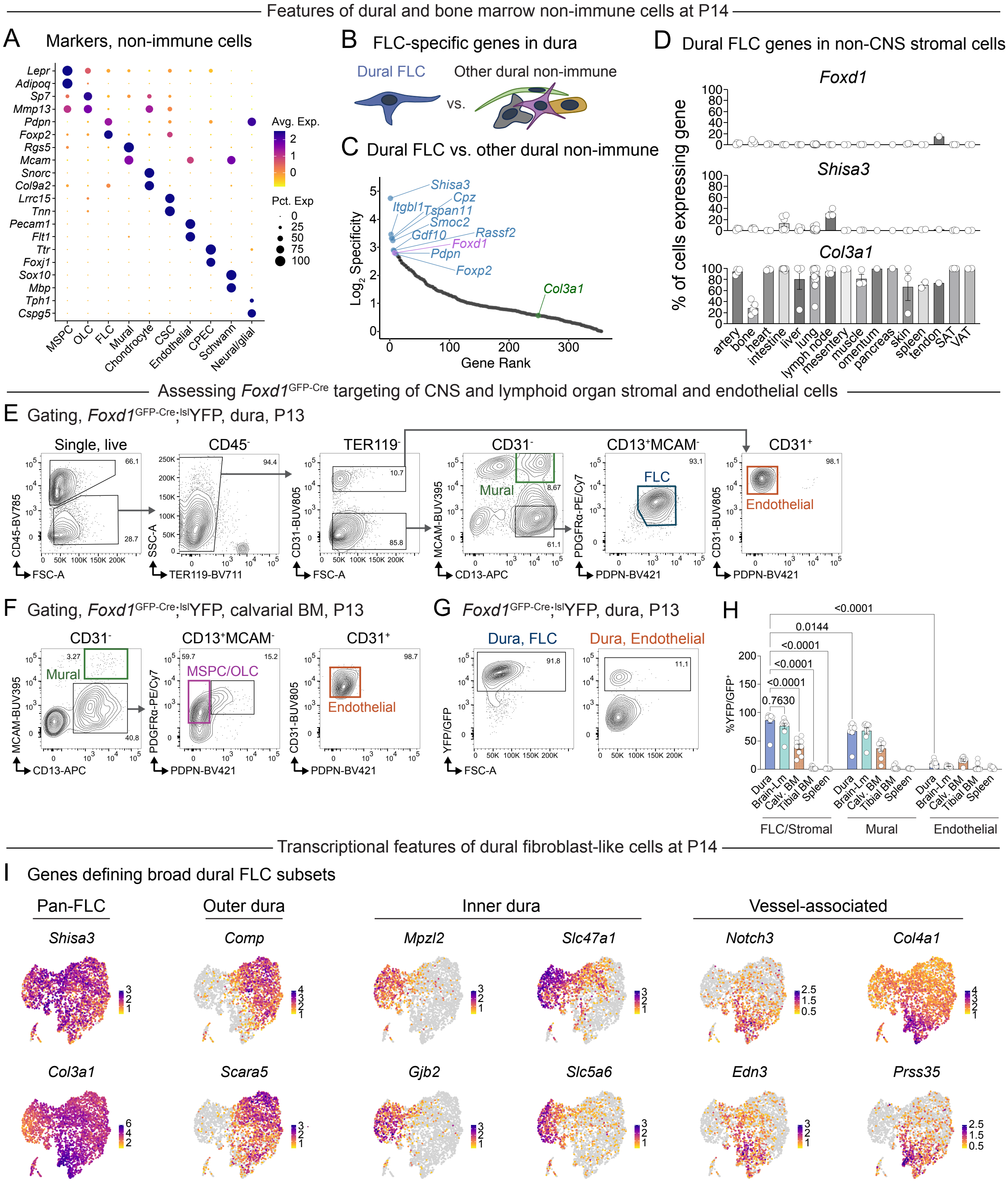

Figure S6. **Molecular characterization and targeting of CNS stromal niches**

(A) Markers defining non-immune cell subtypes across the dura, calvarial bone marrow, and tibial bone marrow at P14 (related to Figure 5A). MSPC—Mesenchymal stem and progenitor cell; OLC—Osteolineage cell; FLC—Fibroblast-like cell; Mural cell (smooth muscle cell and pericyte); Chondrocyte; CSC—Cranial stromal cell; Endothelial cell; Schwann cell; Neural/glial cell (pinealocyte, astrocyte-like cell, neuronal cell); CPEC—Choroid plexus epithelial cell.

bioRxiv preprint doi: https://doi.org/10.1101/2025.11.27.690844; this version posted November 29, 2025. The copyright holder for this preprint (which was not certified by peer review) is the author/funder, who has granted bioRxiv a license to display the preprint in perpetuity. It is made available under aCC-BY 4.0 International license.

(B) Experimental design for identification of genes enriched in and specific for dural FLCs compared to other non-immune cells in the dura. Differentially expressed genes upregulated in dural FLCs were assigned a specificity score where specificity = % expression in FLCs/% expression in other dural non-immune cells. This list was further filtered for genes expressed in at least 70% of dural FLCs.

(C) Dural FLC-enriched genes, ranked by specificity and plotted on a $\log_2$ scale. The top 10 genes specific to dural FLCs are labeled (top 10, blue; gene of interest *Foxd1*, purple), as well as the global fibroblast marker *Col3a1* (green).

(D) Expression of dural FLC-enriched genes *Foxd1* and *Shisa3* and global fibroblast marker *Col3a1* in fibroblasts and other stromal populations across the body. Data is derived from FibroXplorer and includes a compilation of scRNA-seq datasets describing fibroblasts across steady state mouse organs. For each organ, the percentage of fibroblasts/stromal cells expressing the candidate gene at a level > 0 was calculated and plotted. SAT—subcutaneous adipose tissue; VAT—visceral adipose tissue.

(E) Representative gating strategy to identify stromal and endothelial cell populations in *Foxd1*$^{GFP\text{-}Cre}$;^{lsl}YFP mice at P13-P14. The dura is shown here and is representative of gating for pooled brain and leptomeninges, and spleen, samples. After gating live, single, CD45$^-$, TER119$^-$, cells, endothelial cells were identified as CD31$^+$PDPN$^-$. CD31$^-$ cells were divided into CD13$^+$MCAM$^+$ (mural) and CD13$^+$MCAM$^-$ groups. In the dura, FLCs were identified from CD13$^+$MCAM$^-$ group as PDGFRα$^+$PDPN$^+$ cells. In the brain-leptomeninges and spleen, FLCs/stromal cells were designated as CD13$^+$MCAM$^-$ due to variable expression of PDGFRα and PDPN.

(F) Representative gating strategy to identify stromal and endothelial cell populations in *Foxd1*$^{GFP\text{-}Cre}$;^{lsl}YFP mice at P13-P14. The calvarial bone marrow (BM) is shown here and is representative of tibial bone marrow gating. After gating live, single, CD45$^-$, TER119$^-$, cells, endothelial cells were identified as CD31$^+$PDPN$^-$. CD31$^-$ cells were divided into CD13$^+$MCAM$^+$ (mural) and CD13$^+$MCAM$^-$ groups. CD13$^+$MCAM$^-$ cells were further gated for PDGFRα$^+$PDPN$^-$ cells to identify MSPC/OLC populations.

(G) Representative flow cytometry plots of YFP/GFP expression in dural FLCs and dural endothelial cells P13 in *Foxd1*$^{GFP\text{-}Cre}$;^{lsl}YFP mice. Note that this fluorescent signal reflects a combination of the recombined YFP allele and some *Foxd1*-driven GFP from the Cre line.

(H) Percentage of stromal and endothelial populations from P13-P14 *Foxd1*$^{GFP\text{-}Cre}$;^{lsl}YFP mice expressing YFP/GFP, indicating targeting by *Foxd1*-driven Cre. Note that this fluorescent signal reflects a combination of the recombined YFP allele and some *Foxd1*-driven GFP from the Cre line. FLC/Stromal (dural FLCs, pooled brain-leptomeningeal (Lm) FLCs, calvarial bone marrow (Calv. BM) and tibial bone marrow MSPCs/OLCs, and spleen stromal cells); MCAM$^+$CD13$^+$ mural cells from all organs; and CD31$^+$PDPN$^-$ blood endothelial cells from all organs. n = 9 mice pooled across three independent experiments. Error bars indicate SEM. Two-way ANOVA with Tukey's multiple comparisons test. Cell type x Tissue interaction: $F(8, 120) = 33.10$, $P < 0.0001$. Select post-hoc tests are displayed comparing dural FLCs to other relevant cell types.

(I) Additional genes defining broad groups of dural FLCs (outer dura, inner dura, vessel-associated dura), corresponding to Figure 5H.

bioRxiv preprint doi: https://doi.org/10.1101/2025.11.27.690844; this version posted November 29, 2025. The copyright holder for this preprint (which was not certified by peer review) is the author/funder, who has granted bioRxiv a license to display the preprint in perpetuity. It is made available under aCC-BY 4.0 International license.

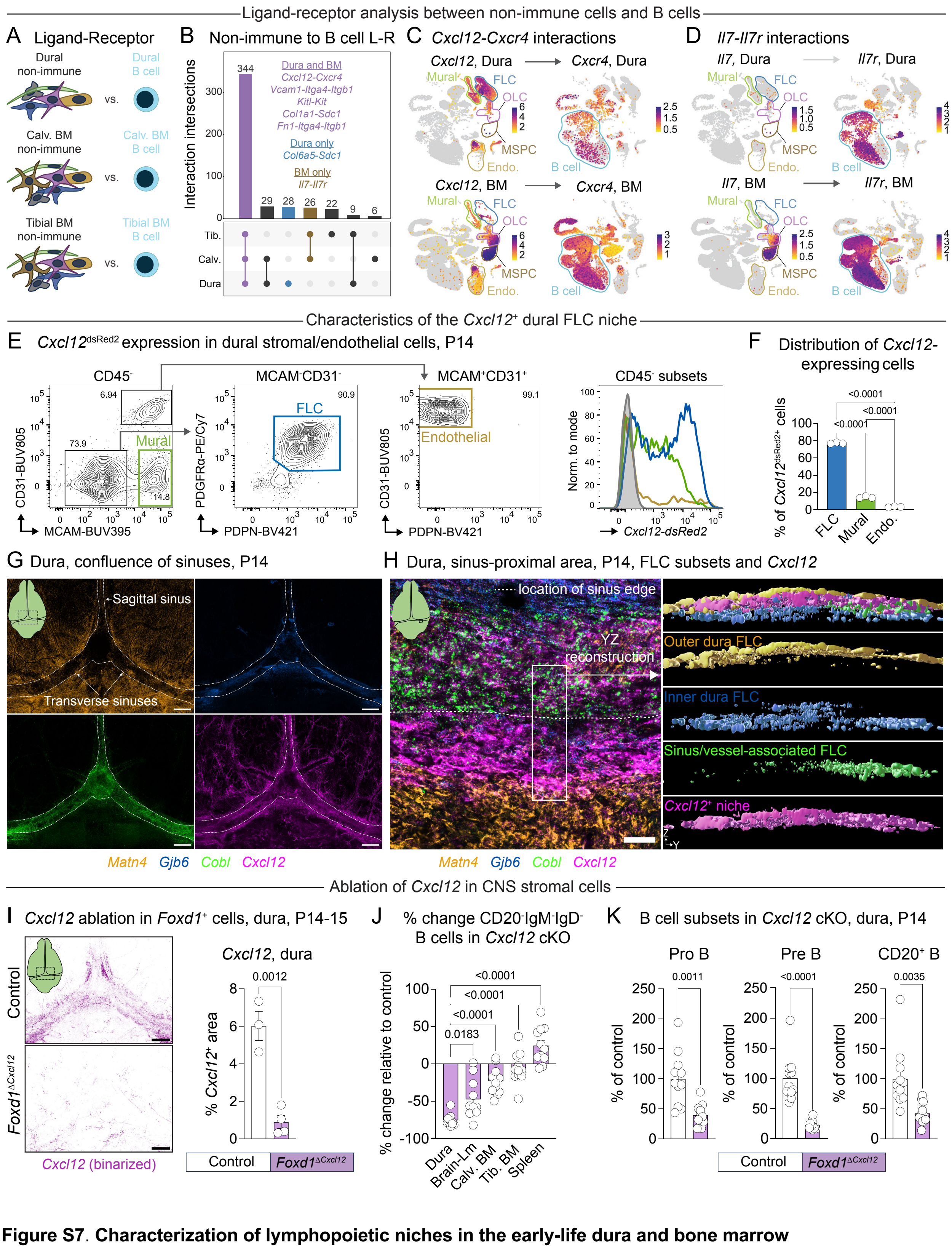

Figure S7. Characterization of lymphopoietic niches in the early-life dura and bone marrow

(A) Ligand-receptor interactions between non-immune cells and B cells were calculated for the dura, calvarial bone marrow (Calv. BM), and tibial bone marrow separately using LIANA.

bioRxiv preprint doi: https://doi.org/10.1101/2025.11.27.690844; this version posted November 29, 2025. The copyright holder for this preprint (which was not certified by peer review) is the author/funder, who has granted bioRxiv a license to display the preprint in perpetuity. It is made available under aCC-BY 4.0 International license.

(B) Intersection of ligand-receptor interactions between non-immune cells and B cells in the dura, calvarial bone marrow (Calv.), and tibial bone marrow (Tib.), from analysis described in (A). Numbers of shared versus unique non-immune cell–to–B cell interactions plotted across organs and select shared or unique interactions are highlighted.

(C) Expression of *Cxcl12* and cognate receptor *Cxcr4* in dural or bone marrow (BM) non-immune and immune cells at P14. FLC—Fibroblast-like cell; OLC—Osteolineage cell; MSPC—Mesenchymal stem and progenitor cell; Endo.—Endothelial cell; Mural cell (pericyte and smooth muscle cell); B cell.

(D) Expression of *Il7* and cognate receptor *Il7r* in dural or bone marrow (BM) non-immune and immune cells at P14. FLC—Fibroblast-like cell; OLC—Osteolineage cell; MSPC—Mesenchymal stem and progenitor cell; Endo.—Endothelial cell; Mural cell (pericyte and smooth muscle cell); B cell.

(E) Flow cytometric identification of stromal and endothelial subsets in *Cxcl12*^{dsRed2} mice at P14. Live, single, CD45$^-$ cells were further gated to identify endothelial cells (MCAM$^+$CD31$^+$PDPN$^-$), mural cells (MCAM$^+$CD31$^-$), and FLCs (MCAM$^-$CD31$^-$PDGFRα^+PDPN$^+$). A histogram of *Cxcl12*^{dsRed2} expression in each cell type is shown compared to CD45$^-$ cells from a dsRed2-negative littermate control. DsRed2-negative cells, grey; endothelial cells, gold; mural cells, green; FLCs, blue.

(F) Percent of total CD45$^-$, *Cxcl12*^{dsRed2} expressing cells in the P14 dura that are FLCs, mural cells, or endothelial cells. n = 3 littermates from one experiment. Error bars indicate SEM. One-way ANOVA with Tukey's multiple comparisons test. F (2, 6) = 5667, P < 0.0001.

(G) Representative confocal tile scan of the confluence of the sinuses at P14 depicting the localization of *Cxcl12* and FLC subsets. *Matn4*—outer dura, orange; *Gjb6*—inner dura, blue; *Cobl*—sinus/perivascular dura, green, *Cxcl12*, magenta. White outlines indicate the approximate location of the dural sinuses based on *Cobl* expression. Image represents a maximum intensity Z projection across the depth of the tissue. Scale bar indicates 500 μm.

(H) Localization of *Cxcl12* and dural FLC subsets composing the outer dura (*Matn4*), inner dura (*Gjb6*), and sinus/vessel-associated dura (*Cobl*). Image represents a maximum intensity projection of 3 μm from a confocal Z stack localized to an area near the transverse sinus (left). Scale bar represents 50 μm. The full Z stack was used to reconstruct this region in 3D using Imaris. Surfaces were constructed out of each channel and then used to visualize the location of *Cxcl12* signal relative to FLC subtypes in the YZ dimension (right).

(I) Representative tile scan images depicting *Cxcl12* expression at the confluence of the dural sinuses in control (*Cxcl12*$^{fl/fl}$) or *Foxd1*$^{\Delta Cxcl12}$ (*Foxd1*$^{GFP\text{-}Cre/+}$;*Cxcl12*$^{fl/fl}$) mice at P14 (left). Quantification of *Cxcl12* signal (right). Expression of *Cxcl12* exon 2, which is flanked by loxP sites in this strain, was visualized by BaseScope. *Cxcl12* signal was binarized before quantification. n = 3 Control and n = 4 *Foxd1*$^{\Delta Cxcl12}$ mice from two litters each containing Control and *Foxd1*$^{\Delta Cxcl12}$ pups at P14-15. Error bars indicate SEM. Student's T test, unpaired, two-tailed.

(J) Flow cytometric quantification of developing (CD19$^+$CD20$^-$IgM$^-$IgD$^-$) B cells across organs at P14 in *Foxd1*$^{\Delta Cxcl12}$ mice, related to Figure 6F. n = 11 control and 11 *Foxd1*$^{\Delta Cxcl12}$ mice pooled from two independent experiments each consisting of one litter containing Control and *Foxd1*$^{\Delta Cxcl12}$ littermates. Percent change in B cell counts in *Foxd1*$^{\Delta Cxcl12}$ mice relative the Control group is plotted for each organ. Error bars indicate SEM. Welch's ANOVA with Dunnett's T3 multiple comparisons test. F (4.000, 23.07) = 67.33, P < 0.0001. Select post-hoc comparisons shown. Brain-Lm—pooled brain and leptomeninges; Calv. BM—calvarial bone marrow; Tib. BM—Tibial bone marrow.

(K) Flow cytometric quantification of dural B cell subsets at P14 in control and *Foxd1*$^{\Delta Cxcl12}$ mice. Within CD19$^+$CD20$^-$IgM$^-$IgD$^-$ cells, Pro B cells were identified as cKit$^+$CD24^{lo} and Pre B cells as cKit$^-$CD24^{hi}. CD20$^+$ B cells were identified as CD19$^+$CD20$^+$. n = 11 control and 11 *Foxd1*$^{\Delta Cxcl12}$ mice pooled from two independent experiments each consisting of one litter containing Control and *Foxd1*$^{\Delta Cxcl12}$ littermates. For each experiment, B cell counts were normalized to the mean count for the control group. Error bars indicate SEM. Welch's t test, two-tailed.
